# ILC2s Control Microfilaremia During *Litomosoides sigmodontis* Infection in *Rag2^-/-^* Mice

**DOI:** 10.3389/fimmu.2022.863663

**Published:** 2022-06-09

**Authors:** Julia J. Reichwald, Frederic Risch, Anna-Lena Neumann, Stefan J. Frohberger, Johanna F. Scheunemann, Benjamin Lenz, Alexandra Ehrens, Wiebke Strutz, Beatrix Schumak, Achim Hoerauf, Marc P. Hübner

**Affiliations:** ^1^ Institute for Medical Microbiology, Immunology and Parasitology, University Hospital Bonn, Bonn, Germany; ^2^ German Center for Infection Research (DZIF), Partner Site Bonn-Cologne, Bonn, Germany

**Keywords:** ILC2, *Litomosoides sigmodontis*, *Rag2^-/-^*, microfilariae, IL-5, T cells, type 2 immune response, filariae

## Abstract

Group 2 innate lymphoid cells (ILC2s) are inducers of type 2 immune responses, but their role during filarial infection remains unclear. In the present study, we used the *Litomosoides sigmodontis* rodent model of filariasis to analyze ILC2s during infection in susceptible BALB/c mice that develop a chronic infection with microfilaremia and semi-susceptible C57BL/6 mice that eliminate the filariae shortly after the molt into adult worms and thus do not develop microfilaremia. ILC2s (CD45^+^ Lineage^-^ TCRβ^-^ CD90.2^+^ Sca-1^+^ IL-33R^+^ GATA-3^+^) were analyzed in the pleural cavity, the site of *L. sigmodontis* infection, after the infective L3 larvae reached the pleural cavity (9 days post infection, dpi), after the molt into adult worms (30dpi) and during the peak of microfilaremia (70dpi). C57BL/6 mice had significantly increased ILC2 numbers compared to BALB/c mice at 30dpi, accompanied by substantially higher IL-5 and IL-13 levels, indicating a stronger type 2 immune response in C57BL/6 mice upon *L. sigmodontis* infection. At this time point the ILC2 numbers positively correlated with the worm burden in both mouse strains. ILC2s and GATA-3^+^ CD4^+^ T cells were the dominant source of IL-5 in *L. sigmodontis*-infected C57BL/6 mice with ILC2s showing a significantly higher IL-5 expression than CD4^+^ T cells. To investigate the importance of ILC2s during *L. sigmodontis* infection, ILC2s were depleted with anti-CD90.2 antibodies in T and B cell-deficient *Rag2^-/-^* C57BL/6 mice on 26-28dpi and the outcome of infection was compared to isotype controls. *Rag2^-/-^* mice were per se susceptible to *L. sigmodontis* infection with significantly higher worm burden than C57BL/6 mice and developed microfilaremia. Depletion of ILC2s did not result in an increased worm burden in *Rag2^-/-^* mice, but led to significantly higher microfilariae numbers compared to isotype controls. In conclusion, our data demonstrate that ILC2s are essentially involved in the control of microfilaremia in *Rag2^-/-^* C57BL/6 mice.

## Introduction

Filarial nematodes can lead to debilitating neglected tropical diseases, including lymphatic filariasis (LF), which often manifests as lymphedema in limbs (elephantiasis) and in the scrotum (hydrocele), and onchocerciasis (river blindness), which causes severe dermatitis and in the worst case leads to blindness (1). LF and onchocerciasis represent a major public health problem in endemic regions due to the chronic nature of the diseases and accompanied socio-economic problems, as people suffering from these diseases are stigmatized and often not able to work (2, 3).

During filarial infections, the host develops protective type 2 immune responses that are mainly characterized by an increase in type 2 cytokines like IL-4, IL-5 and IL-13 and an increase of eosinophils as well as alternatively activated macrophages (4, 5). Indeed, patients that do not develop patent infections, i.e. lack the progeny of the filariae, the microfilariae, were shown to have higher parasite-specific IL-5 levels and a stronger adaptive immune response than patients who were microfilariae-positive (6). To reduce these protective immune responses, filariae modulate the immune system of their hosts and establish over time an immunotolerant milieu to ensure their own survival and reproduction, which also limits the development of pathologies (7).

To investigate protective immune responses to filariae, the *Litomosoides sigmodontis* rodent model was successfully used in the past (8). In BALB/c mice, *L. sigmodontis* can complete its entire life cycle and around 50% of mice become microfilariae-positive, which is comparable to human infections with filariae that cause LF. In contrast, C57BL/6 mice are semi-susceptible for the infection with *L. sigmodontis*, as they eliminate the filariae shortly after the molt into adult filariae and before the onset of microfilaremia (9). This difference in susceptibility of C57BL/6 and BALB/c mice to the infection with *L. sigmodontis* offers the opportunity to identify protective immune responses to filarial infections (10). In the present study we focused on the investigation of group 2 innate lymphoid cells (ILC2s) during *L. sigmodontis* infection. ILCs have been characterized as a distinct immune cell population that, despite their lymphoid nature, are part of the innate immune system (11). ILCs share hereby many of their functions with T cells and can be classified into three distinct groups based on their transcription factors and signature cytokines: ILC1s, ILC2s and ILC3s (12). ILC2s produce type 2 cytokines such as IL-5 and IL-13 and are characterized as CD45^+^ Lineage^-^ TCRβ^-^ CD90.2^+^ Sca-1^+^ IL-33R^+^ GATA-3^+^ (11–13). As they present an important component of the type 2 immune response due to their resemblance to Th2 cells in their functions and phenotype, as well as their interaction with eosinophils and alternatively activated macrophages (14), they are of particular interest in helminth infections. ILC2s were already demonstrated to enhance protective immune responses in mouse models using the intestinal nematodes *Nippostrongylus brasiliensis* and *Heligmosomoides polygyrus* (15, 16). However, their importance during extraintestinal nematode infections has so far not been analyzed in detail and only one study described an increase of ILC2s during *L. sigmodontis* infection in BALB/c mice (17). Given that both IL-5 and eosinophils have previously been shown to be essential for the clearance of adult worms and microfilariae during *L. sigmodontis* infection (18) and ILC2s are associated with both, the aim of the present study was to compare the kinetics of ILC2s during *L. sigmodontis* infection in susceptible BALB/c and semi-susceptible C57BL/6 mice. Subsequently, ILC2s of both mouse strains were examined with respect to their IL-5 expression and finally depletion experiments were performed in T and B cell-deficient *Rag2^-/-^* mice to determine whether ILC2s contribute to protective immune responses against *L. sigmodontis*.

## Materials and Methods

### Animals

All experiments were performed with 6 to 8-week-old female BALB/c and C57BL/6 mice that were purchased from Janvier Labs, Saint-Berthevin, France and kept in individually ventilated cages within the animal facility at the Institute for Medical Microbiology, Immunology and Parasitology (IMMIP), University Hospital Bonn. *Rag2^-/-^* mice (19) were kindly provided by Dr. Isis Ludwig-Portugall from the Institute for Experimental Immunology, University Hospital Bonn, and bred in the animal facility of the IMMIP, University Hospital Bonn. All animal experiments were performed according to the EU Directive 2010/63/EU and were approved by the state authorities (AZ 84-02.04.2016.A331 and AZ 81-02.04.2020.A103, Landesamt für Natur, Umwelt und Verbraucherschutz, Recklinghausen, Germany). Water and food were provided *ad libitum*. Animals were checked daily for wellbeing. *L. sigmodontis*-infected animals were additionally scored once per week (score A to C) by measuring body weight and analysing their behaviour and appearance. Experimental mice were analyzed at 9, 30, 63 and 70 days after infection.

### Natural *Litomosoides sigmodontis* Infection

Mice were naturally infected with *L. sigmodontis* as previously described (20). Briefly, the mice were exposed to *L. sigmodontis*-infected tropical rat mites (*Ornithonyssus bacoti*) for 24h, which transmitted with their bite the infective L3 larvae. All mice of one experiment were exposed to the same population of mites.

### Isolation of Worms and Pleural Cavity Cells

Mice were euthanized with an overdose isoflurane (Abbvie, Wiesbaden, Germany). The pleural cavity was washed with 5ml cold and sterile PBS using a Pasteur pipette (Ratiolab GmbH, Dreieich, Germany). The fluid was filtered through cell gaze (41µm, Labomedic, Bonn, Germany) to isolate the filariae. The first ml was centrifuged separately and the supernatant was stored at -20°C for cytokine measurement. Isolated cells were centrifuged for 5 min at 4°C and 400g. Afterwards, pellets were resuspended in 500µl RBC lysis buffer (eBioscience by Thermo Fisher Scientific, Waltham, USA) and the reaction was stopped with 5 ml sterile MACS buffer (1x PBS, 1% FBS (PAN Biotech, Aidenbach, Germany), 2mM EDTA (Roth, Karlsruhe, Germany). Cells were centrifuged again, resuspended in 2ml sterile PBS and counted using CASY TT (Schärfe Systems, Reutlingen, Germany).

### Analysis of Worm Burden, Microfilarial Load and Female Embryogenesis

Filariae were collected during pleura lavage and placed in a 6-well plate (Greiner bio-one GmbH, Kremsmüster, Austria) containing 1x PBS. Filariae from each mouse were identified as female and male filariae by checking for the vulva of female worms and the spiculae of male worms using a light microscope. Filariae were counted and measured using a ruler.

For determination of the microfilarial load in peripheral blood, 50µl of blood were taken from the animals *via* the *Vena facialis* on day 50, 57, 63 and 70 after *L. sigmodontis* infection using a lancet (4 mm, Braintree Scientific, Braintree, USA). The blood was collected in an EDTA tube (Sarstedt AG & Co. KG, Nümbrecht, Germany) and mixed with 950µl RBC lysis buffer (eBioscience by Thermo Fisher Scientific, Waltham, USA), incubated for 5 min at room temperature (RT) and centrifuged for 5 min at 400g and RT. The whole pellet was transferred to a glass slide (Engelbrecht Medizin- und Labortechnik GmbH, Edermünde, Germany) and microfilariae were counted using a microscope.

In order to analyze female embryogenesis, up to 5 female worms per mouse were placed in 4% formaldehyde (Sigma-Aldrich, München, Germany) diluted in 1x PBS for 24h. Afterwards, formaldehyde was removed and replaced with 60% ethanol. Samples were stored at RT until analysis. Worms were homogenized in 80µl PBS and 20µl Hinkelmann solution (0.5% eosin Y, 0.5% phenol, 0.185% formaldehyde in dH_2_O) using a BioMasher. 1:10 and 1:100 dilutions in Hinkelmann solution were prepared if necessary. Embryonic stages (egg, morula, pretzel, stretched microfilaria) were determined and enumerated in 10µl under a light microscope.

### Depletion of ILC2s

*L. sigmodontis*-infected *Rag2^-/-^* mice were treated intraperitoneally (i.p.) with 500µg anti-CD90.2 (*InVivo*MAb anti-mouse Thy1.2 (CD90.2), clone 30H12 by BioXCell, Lebanon, USA) or 500µg isotype control (*InVivo*MAb rat IgG2b isotype control, anti-keyhole limpet hemocyanin, clone LTF-2 by BioXCell) in 200µl PBS at day 26, 27, and 28 post infection. *L. sigmodontis*-infected C57BL/6 mice served as controls. *Ex vivo* analyses were performed 63dpi.

### Cytokine Measurement

For determination of cytokine concentrations (IL-4, IL-5, IFN-γ and IL-13) in the pleural cavity lavage, the Ready-SET-Go! ELISA kits by eBioscience (Thermo Fisher Scientific, Waltham, USA) were used according to the manufacturer’s instructions. All plates were read at 450 nm and 570 nm with Spectramax 190 by subtracting the values of the latter wavelength from the first.

### Restimulation of Pleural Cavity Cells

For intracellular staining of IL-5, 1x10^6^ pleural cavity cells per mouse were stimulated with 1x eBioscience Cell Stimulation Cocktail plus protein transport inhibitors (Invitrogen, Carlsbad, USA) in 200µl complete IMDM medium (IMDM (Gibco by life technologies corporation, Carlsbad, USA) including 10% FBS, 1% Penicillin/Streptomycin, 1% L-Glutamine (Gibco by life technologies corporation, Carlsbad, USA) for 3h at 37°C before flow cytometric staining.

### Flow Cytometry

For flow cytometry analysis of pleural cavity cells without intracellular cytokine staining, 1x10^6^ pleura lavage cells per mouse were directly used for the staining procedure. For intracellular staining, cells were centrifuged for 5 min at 4°C and 400g and resuspended in 200µl Fixation/Permeabilization (Foxp3 Transcription Factor Staining Buffer Kit by Invitrogen, Carlsbad, USA). After 20 min at RT, cells were centrifuged again, 200µl of blocking buffer (PBS/1% BSA + 1:1000 Rat IgG (Sigma-Aldrich, St. Louis, USA)) were added and the cells were incubated overnight at 4°C. Afterwards the cells were centrifuged and incubated with 200µl Permeabilization buffer (Foxp3 Transcription Factor Staining Buffer Kit) for 20 min at RT. Antibodies were incubated with cells for 45 min at 4°C, cells were washed twice with 150µl Permeabilization buffer and were subsequently resuspended in 150µl MACS buffer before measurement. The following fluorescently-labelled specific antibodies for flow cytometric analysis were purchased from BioLegend, San Diego, CA, USA, if not stated otherwise: anti-mouse CD11b (BV510, clone M1/70), CD19 (APC, eBio1D3 by eBioscience), CD4 (BV605, clone RM4-5), CD45 (FITC, clone 30-F11), CD8 (PerCP-Cy5.5, clone 53-6.7), CD90.2 (PE, clone 53-2.1), GATA-3 (PE-Cy7, clone L50-823, BD Biosciences), IL-33R (APC, clone DIH9), IL-5 (PE, clone TRFK5, eBioscience by Thermo Fisher Scientific), Lineage cocktail (Pacific Blue, includes anti-mouse CD3, clone 17A2; anti-mouse Ly-6G/Ly-6C, clone RB6-8C5; anti-mouse CD11b, clone M1/70; anti-mouse CD45R/B220, clone RA3-6B2; anti-mouse TER-119/erythroid cells, clone Ter-119), Ly6C (APC-Cy7, clone HK1.4), Ly6G (BV421, clone 1A8), NKp46 (PE, clone 29A1.4), Sca-1 (BV510, clone D7), Siglec-F (PE, APC-Cy7 clone E50-2440 by BD Biosciences), T-bet (APC, clone 4B10), TCRβ (Alexa Fluor 700, clone H57-597).

### Statistics

Data analysis was performed by using GraphPad Prism 8 (GraphPad Software, San Diego, USA). For non-parametric comparison of three or more groups Kruskal-Wallis-test with Dunn’s post-test was used. For comparison of two groups Mann-Whitney-test was used. For correlation analysis, Spearman correlation was used. Spearman test for heteroscedasticity was performed and only data from multiple experiments failing the test for heteroscedasticity were pooled. For the analysis of microfilariae over time, mixed-effects analysis or Two-Way ANOVA with Bonferroni multiple comparison test was performed. Error bars represent the median with interquartile ranges. A p-value of <0.05 was considered to be statistically significant.

## Results

### C57BL/6 Mice Eliminate *L. sigmodontis* Infection, Whereas BALB/c Mice Develop Chronic Infections

**Figure 1:**
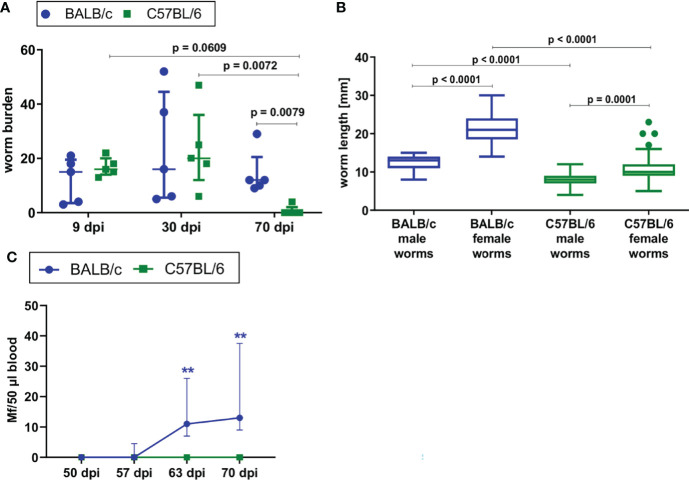
BALB/c mice develop chronic *L. sigmodontis* infection, whereas C57BL/6 mice clear the infection after the molt into adult filariae. **(A)** Worm burden of naturally *L. sigmodontis*-infected susceptible BALB/c mice and semi-susceptible C57BL/6 mice 9, 30 and 70 days after infection (dpi). **(B)** Worm length [mm] of male and female filariae at 30dpi. **(C)** Microfilariae counts in peripheral blood over time. **(A, C)** n = 5, data are representative for 2 (9dpi) or 4 (30, 70dpi) independent experiments. B: n = 49-67 worms, data are representative for 4 independent experiments. **(A)** Data shown as median with IQR; Kruskal-Wallis with Dunn’s post-test. **(B)** Data shown as Box and Whiskers with Tukey; Kruskal-Wallis with Dunn’s post-test. **(C)** 2-way ANOVA and Bonferroni multiple comparison test. **p < 0.01 Mf: Microfilariae.

To confirm previous findings in literature, *L. sigmodontis* infection was directly compared in susceptible BALB/c and semi-susceptible C57BL/6 mice upon natural *L. sigmodontis* infection *via* exposure to the mite vector. BALB/c mice developed a chronic *L. sigmodontis* infection, while *L. sigmodontis* adult filariae were eliminated by day 70 in C57BL/6 mice (**Figure 1A**). The worm burden was comparable between both mouse strains at day 9 and 30 of *L. sigmodontis* infection, while it was significantly lower in C57BL/6 mice than in BALB/c mice 70dpi (p=0.0079). No worms were detected in C57BL/6 mice at that time point, except for one mouse, which had four worms left. As an additional parameter, length of the filariae were determined at 30dpi, a time point where the majority of filariae completed the molt into the adult stage. At 30dpi, the length of male and female worms of C57BL/6 mice was significantly lower compared to the length of BALB/c mice (p=0.0001, **Figure 1B**), which may be due to the limited susceptibility of C57BL/6 mice to *L. sigmodontis* infection. BALB/c mice developed microfilaremia by day 63 (100% of BALB/c mice were microfilariae positive), while none of the infected C57BL/6 mice had detectable microfilariae in the peripheral blood (**Figure 1C**). Thus, BALB/c mice are fully susceptible to *L. sigmodontis* infection, whereas C57BL/6 are semi-susceptible and efficiently eliminate the parasite.

### ILC2s and Type 2 Immune Responses Are Enhanced in *L. sigmodontis*-Infected C57BL/6 Mice in Comparison to BALB/c Mice and Positively Correlate With the Worm Burden

In order to correlate susceptibility to *L. sigmodontis* infection with ILC2 responses, ILC2s were quantified during the course of *L. sigmodontis* infection by flow cytometry in the pleural cavity, the site of filarial infection, and IL-4, IL-5, IL-13 and IFNγ levels were measured in the pleural lavage. ILC2s were characterized as CD45^+^ Lineage^-^ TCRβ^-^ CD90.2^+^ Sca-1^+/-^ IL-33R^+^ GATA-3^+^ (**S1 Figure A**).

**Figure 2:**
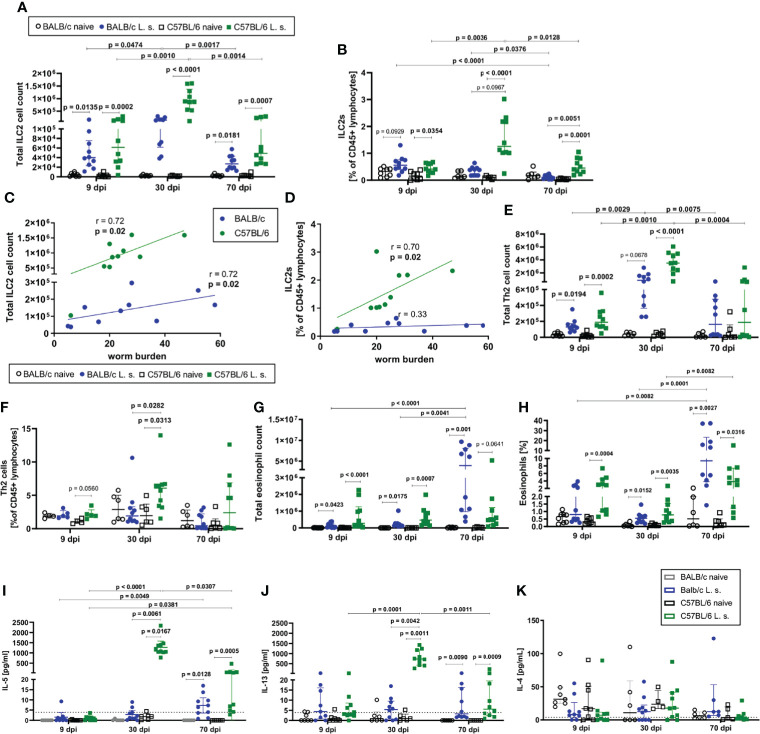
ILC2s and type 2 immune responses are increased in C57BL/6 mice compared to BALB/c mice. **(A)** Total ILC2 cell count and **(B)** ILC2s proportion [% of CD45^+^ lymphocytes] in the pleural cavity of naturally *L. sigmodontis*-infected susceptible BALB/c and semi-susceptible C57BL/6 mice 9, 30 and 70 days after infection (dpi). Correlation of **(C)** pleural cavity ILC2 cell counts and **(D)** pleural cavity ILC2 frequencies [% of CD45^+^ lymphocytes] with the worm burden at 30dpi in BALB/c and C57BL/6 mice. **(E)** Total Th2 cell count and **(F)** Th2 cell frequency [% of CD45^+^ lymphocytes] in the pleural cavity. **(G)** Total eosinophil count and **(H)** eosinophil frequency [% of pleural cavity cells]. **(I)** IL-5, **(J)** IL-13 and **(K)** IL-4 levels in the pleural cavity lavage. **(A–D)** n = 6-10 mice, pooled data from 2 independent experiments per time point. **(E–H)** n = 6-10 mice, pooled data from 2 independent experiments per time point. **(A, B, E–K)** Data shown as median with IQR; Kruskal-Wallis with Dunn’s post-test. **(C, D)** Correlation analysis was performed by nonparametric Spearman correlation. Spearman’s test for heteroscedasticity was performed and only data failing the heteroscedasticity were pooled.

ILC2 cell counts in the pleural cavity as well as frequencies among CD45^+^ lymphocytes increased 9dpi in both BALB/c and C57BL/6 mice compared to naïve controls (**Figures 2A, B**). At 30dpi, ILC2 cell counts and frequencies further increased significantly only in C57BL/6 mice (p<0.0001) in comparison to C57BL/6 mice at 9dpi (**Figure 2A**), resulting also in a trend to higher frequency of ILC2s in C57BL/6 mice than in BALB/c mice at 30dpi (p=0.0967) (**Figure 2B**). While the ILC2 cell counts and proportions peaked at 30dpi in C57BL/6 mice, ILC2 cell counts in both strains and frequencies in C57BL/6 mice were still significantly increased at 70dpi in comparison to the naïve controls (ILC2 cell counts: BALB/c (p=0.0181), C57BL/6 (p=0.0007); ILC2 cell frequencies: BALB/c (p>0.9999), C57BL/6 (p=0.0001) (**Figure 2A**). Although the majority of C57BL/6 mice cleared the adult worm burden by 70dpi (**Figure 1A**), ILC2 cell frequencies were still significantly higher compared to *L. sigmodontis*-infected BALB/c mice (p=0.0051) at that time point. Of note, the increase in ILC2 cell counts and frequencies during *L. sigmodontis* infection was not observed in lungs and both mouse strains showed no differences in lung ILC2 frequencies and total numbers (data not shown). Interestingly, at 30dpi the pleural ILC2 cell count positively correlated with the worm burden in both BALB/c (r=0.72, p=0.02) and C57BL/6 mice (r=0.72, p=0.02) (**Figure 2C**). Frequencies of ILC2s positively correlated in C57BL/6 mice with the worm burden at 30dpi (r=0.70, p=0.02), but not in BALB/c mice (**Figure 2D**). Further analysis of pleural cavity ILC2s revealed a different phenotype of ILC2s in BALB/c and C57BL/6 mice after *L. sigmodontis* infection (**S1 Figure B**). ILC2s of *L. sigmodontis*-infected C57BL/6 mice showed a significantly higher Sca-1 expression than the ILC2s of BALB/c mice at both 30dpi and 70dpi. Furthermore, CD90.2 and IL-33R (ST-2) expression of ILC2s of C57BL/6 mice was significantly higher than in BALB/c mice 70dpi. The analysis of the adaptive counterparts of ILC2s, the Th2 cells (characterized as CD45^+^ Lin^+^ TCRb^+^ CD90.2^+^ GATA-3^+^, **S1 Figure A**), revealed an increase in the total Th2 cell count at 9 and 30dpi in both BALB/c and C57BL/6 *L. sigmodontis*-infected mice compared to naïve controls (**Figure 2E**). The proportion of Th2 cells at 30dpi increased significantly only in C57BL/6 mice (p=0.0313) compared to naïve controls and was significantly higher compared to *L. sigmodontis*-infected BALB/c mice (p=0.0282) (**Figure 2F**). At that time point, ILC2 numbers also positively correlated with Th2 cells in both mouse strains (r=0.6, p=0.08 for C57BL/6 and r=0.87, p=0.002 for BALB/c; data not shown). On day 70 after infection, no differences were observed between BALB/c and C57BL/6 mice regarding Th2 total cell counts and frequencies (**Figures 2E, F**). No significant differences were detected regarding Th1 cells in the pleural cavity when comparing *L. sigmodontis*-infected BALB/c and C57BL/6 mice (**S1 Figure D**).

Since ILC2s interact closely with eosinophils, the kinetics of eosinophils in the pleural cavity were also analyzed (**Figures 2G, H**). In both BALB/c and C57BL/6 mice, a significant increase in the total cell count of eosinophils compared to the respective naïve controls was detected at all time points (9dpi, 30dpi, and 70dpi) (**Figure 2G**), with eosinophil counts being highest in C57BL/6 mice at 9 and 30dpi, and BALB/c mice at 70dpi. Similarly, frequencies of eosinophils in the pleural cavity significantly increased in both mouse strains compared to naive controls 30dpi and 70dpi (**Figure 2H**). Both mouse strains showed a significant increase in eosinophils from 30dpi to 70dpi (p=0.0001 for BALB/c, p=0.0082 for C57BL/6, **Figure 2H**). Similar to eosinophil numbers, eosinophil frequencies were highest in C57BL/6 mice at 9 and 30dpi, and BALB/c mice at 70dpi.

As a next step, the cytokine milieu at the site of infection was evaluated. At day 30 after *L. sigmodontis* infection, IL-5 (p=0.0167) (**Figure 2I**) and IL-13 (p=0.0011) (**Figure 2J**) levels in the pleural cavity lavage increased significantly only in C57BL/6 mice compared to naïve controls, whereas both cytokines were around the detection limit for BALB/c mice. At 70dpi, IL-5 and IL-13 levels were significantly increased in both mouse strains compared to naïve controls, with IL-5 levels still tending to be higher in *L. sigmodontis*-infected C57BL/6 mice than in BALB/c mice. IL-5 and IL-13 levels peaked at day 30 in C57BL/6 mice, as it was observed for ILC2s. For IL-4 neither significant differences were detected between naïve controls and the respective infected mice, nor between *L. sigmodontis*-infected BALB/c and C57BL/6 mice (**Figure 2K**). Moreover, levels of the Th1-associated cytokine IFN-γ increased in the pleural cavity following *L. sigmodontis* infection only in C57BL/6 mice 30dpi but not BALB/c mice leading to significantly higher IFN-γ levels in C57BL/6 mice compared to BALB/c mice (p=0.0316, **S1 Figure E**). IFN-γ significantly decreased from 30dpi to 70dpi in C57BL/6 mice (**S1 Figure E**).

Based on these observations, it is concluded that C57BL/6 mice develop a stronger immune response to *L. sigmodontis* in comparison to BALB/c mice, as was shown for the increase in ILC2s, Th2 cells and type 2 cytokines IL-5 and IL-13 as well as the type 1 cytokine IFN-γ. The type 2 immune response was most prominent in C57BL/6 mice at 30dpi, a time point shortly before the clearance of adult worms occurs in this mouse strain.

### ILC2s Are Potent Producers of IL-5 During *L. sigmodontis* Infection

Next, it was investigated which cells contribute to the high IL-5 levels in the pleural cavity of *L. sigmodontis*-infected C57BL/6 mice and whether ILC2s are involved. For this purpose, intracellular staining of IL-5 in ILC2s and GATA-3^+^ CD4^+^ T cells (Th2 cells) was conducted by flow cytometry (**S2 Figures A, B**).

**Figure 3:**
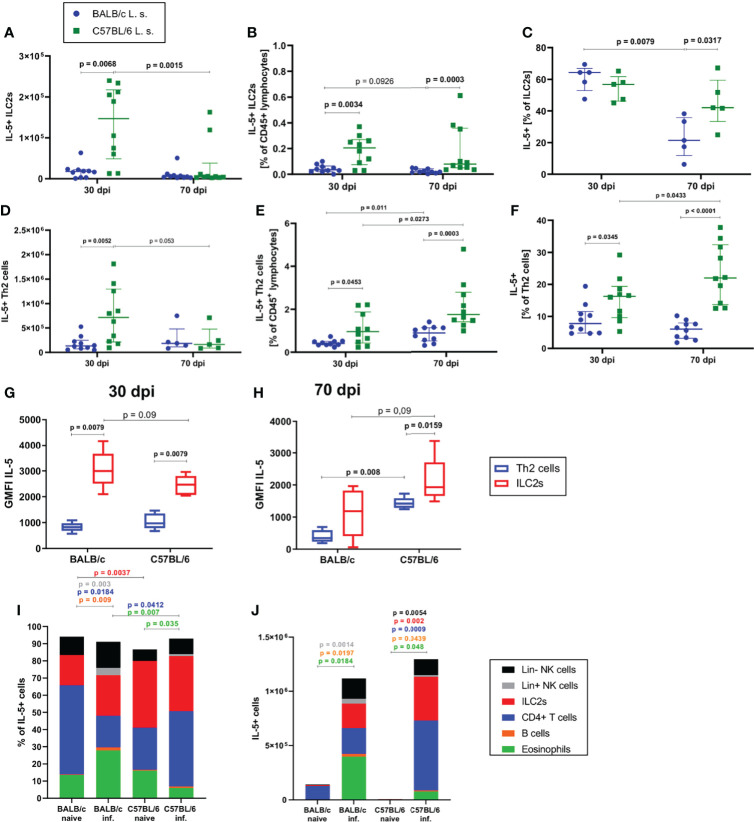
ILC2s are more potent in producing IL-5 than CD4^+^ T cells during *L. sigmodontis* infection. **(A)** Total IL-5^+^ ILC2 cell count in the pleural cavity of naturally *L. sigmodontis*-infected susceptible BALB/c and semi-susceptible C57BL/6 mice at 30 and 70 days after infection (dpi). **(B)** IL-5^+^ ILC2 frequency of CD45^+^ lymphocytes and **(C)** IL-5^+^ ILC2s frequencies of ILC2s in the pleural cavity. **(D)** Total IL-5^+^ Th2 cell count in the pleural cavity of naturally *L. sigmodontis*-infected susceptible BALB/c and semi-susceptible C57BL/6 mice 30 and 70dpi. **(E)** IL-5^+^ Th2 cell frequency of CD45^+^ lymphocytes and **(F)** IL-5^+^ Th2 cells frequency of CD4^+^ T cells in the pleural cavity. IL-5 expression (GMFI) of ILC2s and Th2 cells in 30 day **(G)** and 70 day **(H)** *L. sigmodontis*-infected BALB/c and C57BL/6 mice. **(I)** Composition of IL-5^+^ cells (shown as frequencies) in the pleural cavity of naïve mice and at 30dpi. **(J)** Composition of IL-5^+^ cells (shown as total numbers) in the pleural cavity of naïve mice and at 30dpi. **(A, B, D–F)** n = 10 mice, pooled data from 2 independent experiments per time point. **(C)** n = 5 mice, data shows 1 representative experiment from 2 independent experiments per time point. **(G, H)** n = 5 mice, data shows 1 representative experiment from 2 independent experiments. **(I)** n = 5 mice, data from 1 experiment. **(J)** n = 5 mice, data shows 1 representative experiment from 2 independent experiments for infected groups and 1 experiment for naïve mice. **(A–F)** Data shown as median with IQR, Mann-Whitney test to compare 2 mouse groups per time point and 2 time points per mouse group. **(G, H)** Data shown as a Tukey Box and Whiskers blot, Mann-Whitney test to compare 2 cell types per mouse group, 2 mouse groups per cell type. **(I, J)** Kruskal-Wallis and Dunn’s post-test. Spearman’s test for heteroscedasticity was performed and only data failing the heteroscedasticity were pooled.

Since the strongest differences between BALB/c and C57BL/6 mice were observed at 30 and 70dpi, the following analyses focused on these time points. In line with the observed differences in ILC2 cell numbers and frequencies, there were significantly more IL-5^+^ ILC2s in the pleural cavity of C57BL/6 than in BALB/c mice at 30dpi, both in total cell count (p=0.0068) and in frequency (p=0.0034) (**Figures 3A, B**). Similarly, at 70dpi, C57BL/6 mice had a significantly higher frequency of IL-5^+^ ILC2s than BALB/c mice (p=0.0003, **Figure 3B**). Around 60% of ILC2s in both BALB/c and C57BL/6 mice were positive for IL-5 at 30dpi, but at 70dpi, C57BL/6 mice had a significantly higher frequency of IL-5^+^ ILC2s (40% of ILC2s) in comparison to BALB/c mice (20% of ILC2s) (p=0.0317, **Figure 3C**). A similar outcome was observed for IL-5^+^ GATA3^+^ CD4^+^ T cells. Again, at 30dpi and 70dpi, C57BL/6 mice had significantly more IL-5^+^ Th2 cells than BALB/c mice (**Figures 3D, E**) and significantly more Th2 cells were IL-5^+^ in C57BL/6 mice than in BALB/c mice (p=0.0345 for 30dpi and p<0.0001 for 70dpi) (**Figure 3F**).

With regard to the contribution of IL-5 production by ILC2s and Th2 cells at 30dpi (**Figure 3G**) and 70dpi (**Figure 3H**), the IL-5 expression level of ILC2s was higher than the expression level of Th2 cells in BALB/c mice 30dpi (p=0.0079) and in C57BL/6 mice 30dpi and 70dpi (30dpi: p=0.0079, 70dpi: p=0.0159). At 30dpi, IL-5 expression levels of Th2 cells were comparable between both mouse strains (**Figure 3G**), whereas IL-5 expression levels tended to be lower in ILC2s of C57BL/6 mice in comparison to BALB/c mice (p=0.09). In contrast, at 70dpi, the IL-5 expression levels of ILC2s (p=0.09) and Th2 cells (p=0.008) in C57BL/6 mice were higher than that of the corresponding cells in BALB/c mice (**Figure 3H**). The composition of IL-5^+^ cells also differed between both mouse strains, as is shown in the naïve state as well as at 30dpi. In the naïve state the dominant lymphoid IL-5 source in BALB/c mice were CD4+ T cells whereas in C57BL/6 mice ILC2s presented a significantly higher proportion of IL-5+ cells than in BALB/c mice (p=0.0037, **Figure 3I**). In 30 day *L. sigmodontis*-infected C57BL/6 mice, lymphoid cells in general (ILC2s and CD4^+^ T cells) were the dominant sources of IL-5 (**Figure 3I**), whereas in BALB/c mice eosinophils contributed significantly to the IL-5 production (~30% of IL-5 positive cells) with a significantly higher proportion than in *L. sigmodontis*-infected C57BL/6 mice (p=0.007). Interestingly, at 30dpi, the contribution of CD4^+^ T cells to IL-5 producers was significantly higher in C57BL/6 mice than in BALB/c mice (p=0.03 and p=0.008, respectively) (**Figure 3I**). With regard to the total cell count of IL-5 producers, naïve mice barely showed any IL-5+ cells (**Figure 3J**) and at 30dpi BALB/c and C57BL/6 had comparable numbers of IL-5+ cells in the pleural cavity.

In summary, these results suggest that ILC2s are more potent in producing IL-5 than Th2 cells during *L. sigmodontis* infection, particularly in C57BL/6 mice and thus, may be critical contributors to the cytokine milieu and the resulting type 2 immune response during *L. sigmodontis* infection.

### ILC2 Depletion Enhances the Microfilarial Load in *L. sigmodontis*-Infected T and B Cell-Deficient *Rag2^-/-^* Mice

Given the observed differences in ILC2s during *L. sigmodontis* infection in semi-susceptible C57BL/6 mice in comparison to susceptible BALB/c mice, we next investigated the role of ILC2s in protective immune responses by depletion experiments. Depletion experiments were performed in T and B cell-deficient *Rag2^-/-^* C57BL/6 mice using an anti-CD90.2 depletion antibody. Infection of *Rag2^-/-^* mice resulted in a significantly higher worm burden compared to wild-type C57BL/6 mice 30dpi (**S3 Figure A**) with 100% of the worms being adult in *Rag2^-/-^* mice and 30% in C57BL/6 mice (**S3 Figure B**). Based on those observations, it can be concluded that the adaptive immune system, i.e. T and B cells, is essentially involved in the elimination of *L. sigmodontis*.

**Figure 4:**
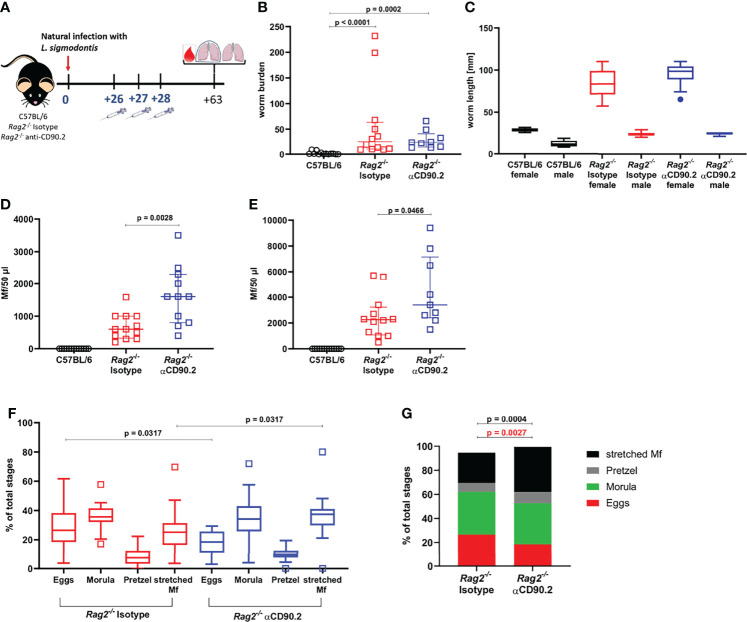
ILC2 depletion enhances the microfilarial load in *L. sigmodontis*-infected *Rag2^-/-^* mice. **(A)** Experimental setup of ILC2 depletion in *Rag2^-/-^* mice. **(B)** Adult worm burden in naturally *L. sigmodontis*-infected C57BL/6 mice, *Rag2^-/-^* mice and ILC2-depleted *Rag2^-/-^* mice at 63dpi. **(C)** Worm length [mm] of female and male worms at 63dpi. **(D)** Microfilariae (Mf) count in 50µl peripheral blood at 57dpi and **(E)** 63dpi. **(F)** Embryonic stages (egg, morula, pretzel, stretched Mf) of female worms 63dpi. **(G)** Distribution of embryonic stages [% of total stages] in female worms 63dpi. **(B, D, E)** n = 11-13 mice, pooled data from 2 independent experiments. **(C)** n = 2 worms for female worms of C57BL/6 mice, n = 6 worms for male worms of C57BL/6 mice, n = 20 worms for female and male worms of Isotype-treated *Rag2^-/-^* mice, n = 14-15 for female and male worms of αCD90.2-treated *Rag2^-/-^* mice; data shows 1 representative experiment from 2 independent experiments. **(F, G)** n = 25-28 female worms per group, data from 1 experiment. **(B)** Data shown as median with IQR, Kruskal-Wallis with Dunn’s post-test. **(C)** Data shown as a Tukey Box and Whiskers blot, Kruskal-Wallis with Dunn’s post-test was used to assess statistical significance. **(D, E)** Data shown as median with IQR, Mann-Whitney test for the comparison of Isotype-treated *Rag2^-/-^* mice with αCD90.2-treated *Rag2^-/-^* mice. **(F)** Data shown as a Tukey Box and Whiskers blot, Kruskal-Wallis with Dunn’s post-test was used to assess statistical significance. **(G)** Mann-Whitney test.

To further investigate the importance of ILC2s, which were significantly reduced in *Rag2^-/-^* mice compared to C57BL/6 mice at 30 dpi (**S3 Figure C**), ILC2s were additionally depleted in *Rag2^-/-^* mice using three injections with anti-CD90.2 from 26-28dpi, shortly before the molt into adult worms (**Figure 4A**). *Rag2^-/-^* mice in general showed a significantly higher worm burden than C57BL/6 mice at 63dpi (**Figure 4B**). However, the depletion of ILC2s in *Rag2^-/-^* mice did not lead to a further increase in worm burden in comparison to the corresponding isotype controls. Similarly, female and male worm length were not affected by the depletion of the ILC2s (**Figure 4C**). Interestingly, ILC2 depletion led to a significant increase in microfilarial load in peripheral blood of *Rag2^-/-^* mice, both at 57dpi (p=0.0028) and 63dpi (p=0.0466) compared to the isotype controls (**Figures 4D, E**). The effect of ILC2 depletion on the microfilarial load was supported by the analysis of the filarial embryogenesis, as the ILC2-depleted mice were characterized by a significantly higher proportion of stretched microfilariae than the isotype controls (p=0.0317) (**Figures 4F, G**). When analyzing the immune cell profile of these mice, ILC2s were detected in low numbers in both isotype-treated and depleted *Rag2^-/-^* mice 63dpi (**S4 Figures A, B**). Their phenotype analyzed by CD90.2 expression was not altered by the depletion (**S4 Figure C**). Moreover, myeloid cells were analyzed (**S4 Figures D–I**). Eosinophils were significantly decreased in numbers (p=0.0302) and frequency (p=0.0013) in depleted *Rag2^-/-^* mice compared to C57BL/6 mice (**S4 Figures D, G**), whereas M2 macrophages were significantly lower in cell count (p=0.0184) and frequency (p=0.003) in *Rag2^-/-^* isotype controls compared to C57BL/6 mice (**S4 Figures E, H**). In contrast, neutrophils were significantly higher in total cell count and frequency in both *Rag2^-/-^* groups compared to C57BL/6 mice (**S4 Figures F, I**). The ILC2 depletion had no significant effect on the myeloid cell populations when comparing isotype controls and depleted mice, although eosinophil frequencies were lowest in depleted *Rag2^-/-^* mice.

In conclusion, our data show that semi-susceptible C57BL/6 mice develop a stronger type 2 immune response during *L. sigmodontis* in comparison to susceptible BALB/c mice, which is associated with a stronger induction of ILC2s, Th2 cells and the type 2 cytokines IL-5 and IL-13 shortly before the elimination of the filariae occurs in C57BL/6 mice. Importantly, ILC2s seem to be crucial to control the microfilarial load in *Rag2^-/-^* C57BL/6 mice and thus affect the susceptibility to *L. sigmodontis* infection.

## Discussion

In the present study, kinetics of ILC2s during *L. sigmodontis* infection in susceptible BALB/c and semi-susceptible C57BL/6 mice were compared and the role of ILC2s in protective immune responses against *L. sigmodontis* infection in *Rag2^-/-^* C57BL/6 mice was determined. In line with previous publications, we confirmed that BALB/c mice develop a chronic *L. sigmodontis* infection, whereas C57BL/6 mice eliminate the infection by 70dpi and do not develop microfilaremia (9, 21, 22). Similar strain-dependent differences in susceptibility to helminth infections are also observed for infections with intestinal helminths such as e.g. *Strongyloides ratti* (23, 24) and *H. polygyrus* (25). However, various studies showed that C57BL/6 mice are susceptible for infections with those intestinal helminths (26, 27), whereas this is not the case for infections with the extraintestinal filarial nematode *L. sigmodontis*.

In general, type 2 immune responses are thought to be protective for both intestinal and extraintestinal helminth infections (5, 8, 10, 18, 28) and C57BL/6 mice usually develop a more prominent type 1 immune response, whereas BALB/c mice are characterized as a rather type 2-dominated mouse strain (29). However, upon *L. sigmodontis* infection, ILC2s numbers were significantly increased in C57BL/6 mice compared to BALB/c mice. In the present study we did not determine, whether this increase in ILC2 numbers during the course of *L. sigmodontis* infection was due to an enhanced proliferation or recruitment of ILC2s. IL-4, which could promote ILC2 proliferation, was not increased in the pleural lavage during *L. sigmodontis* infection. Interestingly, shown increases in ILC2s were specific for the thoracic cavity, as ILC2 numbers and frequencies in lungs of BALB/c and C57BL/6 mice remained comparable at 9, 30 and 70dpi. The increase in ILC2 numbers in the thoracic cavity of C57BL/6 mice was further accompanied by a strong type 2 immune response characterized by high IL-5 levels at the site of infection. Functional analysis revealed that ILC2s had, on a cellular basis, higher levels of IL-5 in comparison to Th2 cells in both mouse strains, and *L. sigmodontis*-infected C57BL/6 mice had significantly more IL-5^+^ Th2 cells and IL-5^+^ ILC2s compared to BALB/c mice at 30dpi. Intriguingly, type 2 immune responses were still increased in C57BL/6 mice after the clearance of the infection at 70dpi in comparison to BALB/c mice, which still harbored filariae at that time point. Thus, our results support previous findings that suggest that protective type 2 immune responses are actively suppressed during chronic *L. sigmodontis* infection in susceptible BALB/c mice, as is indicated by the dependence on important mediators for immunotolerance such as regulatory T cells, IL-10 and PD-1 (30–34).

Given that ILC2s were previously shown to mediate protective immune responses against different intestinal helminths i.e. in *S. ratti* infection through mast cell activation and *N. brasiliensis* infection through involvement in maintenance of alternatively activated macrophages (15, 35), we speculated that ILC2s are also involved in protective immune responses against *L. sigmodontis* infection in semi-susceptible C57BL/6 mice. To investigate the role of ILC2s during *L. sigmodontis* infection by depletion experiments, we used T and B cell-deficient *Rag2^-/-^* C57BL/6 mice. *Rag2^-/-^* mice developed a chronic *L. sigmodontis* infection with a significantly higher worm burden compared to immunocompetent C57BL/6 mice. A similar increased susceptibility to *L. sigmodontis* infection was described for *Rag2IL-2Rγ^-/-^* C57BL/6 mice, which additionally lack NK cells and importantly ILC2s (9). Thus, the adaptive immune system is a crucial part for the elimination of *L. sigmodontis* in C57BL/6 mice with CD4^+^ T cells being important mediators, as shown by previous studies with *L. sigmodontis* (36–38). Additional specific depletion of ILC2s in *L. sigmodontis*-infected *Rag2^-/-^* mice did not increase the recovery of adult worms at 63dpi, but significantly enhanced the microfilarial load in the peripheral blood compared to the isotype controls. Consequently, ILC2s are essential for the control of the microfilarial load in the absence of adaptive immune responses in C57BL/6 mice. Given that eosinophil frequencies were lowest in CD90.2 depleted *Rag2^-/-^* mice and increased in C57BL/6 in comparison to BALB/c mice at 30 dpi, and eosinophils are known to limit microfilaremia (18, 39), we hypothesize that ILC2s contribute to control microfilaremia by triggering type 2 immune responses and eosinophils in special. Future experiments that specifically deplete ILC2s in immunocompetent animals will be interesting to pinpoint the importance of ILC2s in the presence of an intact adaptive immune system during an *L. sigmodontis* infection in both susceptible BALB/c and semi-susceptible C57BL/6 mice, as it can be expected that due to the strong interaction of ILC2s and T cells (40), an even more pronounced effect on the parasitological outcome will be observed.

In summary, the data presented here indicate that semi-susceptible C57BL/6 mice develop a stronger type 2 immune response during *L. sigmodontis* infection in comparison to susceptible BALB/c mice, characterized in particular by a strong increase in ILC2s and IL-5. ILC2s are essential mediators of the immune response to *L. sigmodontis* and depletion of ILC2s in T and B cell-deficient *Rag2^-/-^* mice enhanced the susceptibility to the infection as was shown by an increase in microfilariae numbers in the peripheral blood.

## Data Availability Statement

The original contributions presented in the study are included in the article/**Supplementary Material**. Further inquiries can be directed to the corresponding author.

## Ethics Statement

The animal study was reviewed and approved by Landesamt für Natur, Umwelt und Verbraucherschutz, LANUV, Recklinghausen, Germany.

## Author Contributions

Conceived and designed the experiments: JR, BS, MH. Performed the experiments: JR, FR, A-LN, SF, JS, BL, AE, WS. Analyzed the data: JR, A-LN. Contributed reagents/materials/analysis tools: MH, AH. Wrote the manuscript: JR, MH. All the authors reviewed the manuscript. All authors contributed to the article and approved the submitted version.

## Funding

JR, AE and JS were supported by a PhD scholarship from the Jürgen Manchot Stiftung, Düsseldorf, Germany. JR was supported by BONFOR 2020-7-03. AH and BS are members of the Bonn Cluster of Excellence ImmunoSensation EXC 1023 and AH member of the Bonn Cluster of Excellence ImmunoSensation^2^ EXC 2151. AH and MH are members of the German Center for Infection Research (DZIF). MH received funding from the German Center for Infection Research (TTU 09.701).

## Conflict of Interest

The authors declare that the research was conducted in the absence of any commercial or financial relationships that could be construed as a potential conflict of interest.

## Publisher’s Note

All claims expressed in this article are solely those of the authors and do not necessarily represent those of their affiliated organizations, or those of the publisher, the editors and the reviewers. Any product that may be evaluated in this article, or claim that may be made by its manufacturer, is not guaranteed or endorsed by the publisher.

## References

[1] Taylor MJ Hoerauf A Bockarie M. Lymphatic Filariasis and Onchocerciasis. Lancet (2010) 376(9747):1175–85. doi: 10.1016/S0140-6736(10)60586-7 20739055

[2] World Health Organization. Global Programme to Eliminate Lymphatic Filariasis: Progress Report, 2019. Wkly Epidemiol Rec (2020) 95:509–24. Available at: https://www.who.int/publications/i/item/who-wer9543.

[3] World Health Organization. Elimination of Human Onchocerciasis: Progress Report, 2019–2020. Wkly Epidemiol Rec (2020) 95:545–56. Available at: https://www.who.int/publications/i/item/who-wer9545-545-554.

[4] King CL Nutman TB. Regulation of the Immune Response in Lymphatic Filariasis and Onchocerciasis. Immunol Today (1991) 12(3):A54–8. doi: 10.1016/S0167-5699(05)80016-7 1906280

[5] Babu S Nutman TB. Immunology of Lymphatic Filariasis. Parasite Immunol (2014) 36(8):338–46. doi: 10.1111/pim.12081 PMC3990654 24134686

[6] Arndts K Deininger S Specht S Klarmann U Mand S Adjobimey T. Elevated Adaptive Immune Responses are Associated With Latent Infections of Wuchereria Bancrofti. PLoS Negl Trop Dis (2012) 6(4):e1611. doi: 10.1371/journal.pntd.0001611 22509424 PMC3317915

[7] Maizels RM McSorley HJ. Regulation of the Host Immune System by Helminth Parasites. J Allergy Clin Immunol (2016) 138(3):666–75. doi: 10.1016/j.jaci.2016.07.007 PMC5010150 27476889

[8] Risch F Ritter M Hoerauf A Hübner MP. Human Filariasis-Contributions of the Litomosoides Sigmodontis and Acanthocheilonema Viteae Animal Model. Parasitol Res (2021) 120(12):4125–43. doi: 10.1007/s00436-020-07026-2 PMC8599372 33547508

[9] Layland LE Ajendra J Ritter M Wiszniewsky A Hoerauf A Hübner MP. Development of Patent Litomosoides Sigmodontis Infections in Semi-Susceptible C57BL/6 Mice in the Absence of Adaptive Immune Responses. Parasit Vectors (2015) 8:396. doi: 10.1186/s13071-015-1011-2 26209319 PMC4514938

[10] Finlay CM Allen JE. The Immune Response of Inbred Laboratory Mice to Litomosoides Sigmodontis: A Route to Discovery in Myeloid Cell Biology. Parasite Immunol (2020) 42(7):e12708. doi: 10.1111/pim.12708 32145033 PMC7317388

[11] Artis D Spits H. The Biology of Innate Lymphoid Cells. Nature (2015) 517(7534):293–301. doi: 10.1038/nature14189 25592534

[12] Serafini N Vosshenrich CAJ Di Santo JP. Transcriptional Regulation of Innate Lymphoid Cell Fate. Nat Rev Immunol (2015) 15(7):415–28. doi: 10.1038/nri3855 26065585

[13] Spits H Artis D Colonna M Diefenbach A Di Santo JP Eberl G. Innate Lymphoid Cells–a Proposal for Uniform Nomenclature. Nat Rev Immunol (2013) 13(2):145–9. doi: 10.1038/nri3365 23348417

[14] Jacobsen EA Lesuer WE Nazaroff CD Ochkur SI Doyle AD Wright BL. Eosinophils Induce Recruitment and Activation of ILC2s. J Allergy Clin Immunol (2019) 143(2):AB289. doi: 10.1016/j.jaci.2018.12.885

[15] Bouchery T Kyle R Camberis M Shepherd A Filbey K Smith A. ILC2s and T Cells Cooperate to Ensure Maintenance of M2 Macrophages for Lung Immunity Against Hookworms. Nat Commun (2015) 6:6970. doi: 10.1038/ncomms7970 25912172

[16] Pelly VS Kannan Y Coomes SM Entwistle LJ Rückerl D Seddon B. IL-4-Producing ILC2s are Required for the Differentiation of TH2 Cells Following Heligmosomoides Polygyrus Infection. Mucosal Immunol (2016) 9(6):1407–17. doi: 10.1038/mi.2016.4 PMC5257265 26883724

[17] Boyd A Killoran K Mitre E Nutman TB. Pleural Cavity Type 2 Innate Lymphoid Cells Precede Th2 Expansion in Murine Litomosoides Sigmodontis Infection. Exp Parasitol (2015) 159:118–26. doi: 10.1016/j.exppara.2015.09.006 PMC4679599 26394284

[18] Frohberger SJ Ajendra J Surendar J Stamminger W Ehrens A Buerfent BC. Susceptibility to L. Sigmodontis Infection is Highest in Animals Lacking IL-4r/IL-5 Compared to Single Knockouts of IL-4r, IL-5 or Eosinophils. Parasit Vectors (2019) 12(1):248. doi: 10.1186/s13071-019-3502-z 31109364 PMC6528299

[19] Shinkai Y Rathbun G Lam KP Oltz EM Stewart V Mendelsohn M. RAG-2-Deficient Mice Lack Mature Lymphocytes Owing to Inability to Initiate V(D)J Rearrangement. Cell (1992) 68(5):855–67. doi: 10.1016/0092-8674(92)90029-C 1547487

[20] Frohberger SJ Fercoq F Neumann AL Surendar J Stamminger W Ehrens A. S100A8/S100A9 Deficiency Increases Neutrophil Activation and Protective Immune Responses Against Invading Infective L3 Larvae of the Filarial Nematode Litomosoides Sigmodontis. PLoS Negl Trop Dis (2020) 14:e0008119. doi: 10.1371/journal.pntd.0008119 PMC7064255 32107497

[21] Babayan S Ungeheuer M-N Martin C Attout T Belnoue E Snounou G. Resistance and Susceptibility to Filarial Infection With Litomosoides Sigmodontis are Associated With Early Differences in Parasite Development and in Localized Immune Reactions. Infect Immun (2003) 71(12):6820–9. doi: 10.1128/IAI.71.12.6820-6829.2003 PMC308919 14638768

[22] Petit G Diagne M Maréchal P Owen D Taylor D BAIN O. Maturation of the Filaria Litomosoides Sigmodontis in BALB/c Mice; Comparative Susceptibility of Nine Other Inbred Strains. Ann Parasitol Hum Comp (1992) 67(5):144–50. doi: 10.1051/parasite/1992675144 1295407

[23] Dawkins H Grove DI Dunsmore JD Mitchell GF. Strongyloides Ratti: Susceptibility to Infection and Resistance to Reinfection in Inbred Strains of Mice as Assessed by Excretion of Larvae. Int J Parasitology (1980) 10(2):125–9. doi: 10.1016/0020-7519(80)90023-5 7372395

[24] Hartmann W Blankenhaus B Brunn M-L Meiners J Breloer M. Elucidating Different Pattern of Immunoregulation in BALB/c and C57BL/6 Mice and Their F1 Progeny. Sci Rep (2021) 11(1):1536. doi: 10.1038/s41598-020-79477-7 33452272 PMC7810711

[25] Filbey KJ Grainger JR Smith KA Boon L van Rooijen N Harcus Y. Innate and Adaptive Type 2 Immune Cell Responses in Genetically Controlled Resistance to Intestinal Helminth Infection. Immunol Cell Biol (2014) 92(5):436–48. doi: 10.1038/icb.2013.109 PMC4038150 24492801

[26] McSorley HJ Maizels RM. Helminth Infections and Host Immune Regulation. Clin Microbiol Rev (2012) 25(4):585–608. doi: 10.1128/CMR.05040-11 23034321 PMC3485755

[27] Reitz M Brunn M-L Voehringer D Breloer M. Basophils are Dispensable for the Establishment of Protective Adaptive Immunity Against Primary and Challenge Infection With the Intestinal Helminth Parasite Strongyloides Ratti. PLoS Negl Trop Dis (2018) 12(11):e0006992. doi: 10.1371/journal.pntd.0006992 30496188 PMC6289456

[28] Hörauf A Fleischer B. Immune Responses to Filarial Infection in Laboratory Mice. Med Microbiol Immunol (1997) 185(4):207–15. doi: 10.1007/s004300050032 9138292

[29] Mills CD Kincaid K Alt JM Heilman MJ Hill AM. M-1/M-2 Macrophages and the Th1/Th2 Paradigm. J Immunol (2000) 164(12):6166–73. doi: 10.4049/jimmunol.164.12.6166 10843666

[30] Knipper JA Ivens A Taylor MD. Helminth-Induced Th2 Cell Dysfunction is Distinct From Exhaustion and is Maintained in the Absence of Antigen. PLoS Negl Trop Dis (2019) 13(12):e0007908. doi: 10.1371/journal.pntd.0007908 31815932 PMC6922449

[31] van der Werf N Redpath SA Azuma M Yagita H Taylor MD. Th2 Cell-Intrinsic Hypo-Responsiveness Determines Susceptibility to Helminth Infection. PLoS Pathog (2013) 9(3):e1003215. doi: 10.1371/journal.ppat.1003215 23516361 PMC3597521

[32] Specht S Taylor MD Hoeve MA Allen JE Lang R Hoerauf A. Over Expression of IL-10 by Macrophages Overcomes Resistance to Murine Filariasis. Exp Parasitol (2012) 132(1):90–6. doi: 10.1016/j.exppara.2011.09.003 21959021

[33] Taylor MD Harris A Babayan SA Bain O Culshaw A Allen JE. CTLA-4 and CD4+ CD25+ Regulatory T Cells Inhibit Protective Immunity to Filarial Parasites *In Vivo* . J Immunol (2007) 179(7):4626–34. doi: 10.4049/jimmunol.179.7.4626 17878360

[34] Taylor MD LeGoff L Harris A Malone E Allen JE Maizels RM. Removal of Regulatory T Cell Activity Reverses Hyporesponsiveness and Leads to Filarial Parasite Clearance *In Vivo* . J Immunol (2005) 174(8):4924–33. doi: 10.4049/jimmunol.174.8.4924 15814720

[35] Meiners J Reitz M Rüdiger N Turner J-E Heepmann L Rudolf L. IL-33 Facilitates Rapid Expulsion of the Parasitic Nematode Strongyloides Ratti From the Intestine *via* ILC2- and IL-9-Driven Mast Cell Activation. PLoS Pathog (2020) 16(12):e1009121. doi: 10.1371/journal.ppat.1009121 33351862 PMC7787685

[36] Wiszniewsky A Layland LE Arndts K Wadephul LM Tamadaho RSE Borrero-Wolff D. Adoptive Transfer of Immune Cells Into RAG2IL-2rγ-Deficient Mice During Litomosoides Sigmodontis Infection: A Novel Approach to Investigate Filarial-Specific Immune Responses. Front Immunol (2021) 12:777860. doi: 10.3389/fimmu.2021.777860 34868049 PMC8636703

[37] Finlay CM Parkinson JE Chan BHK Ajendra J Chenery A Morrison A. Genotype and Th2 Cells Control Monocyte to Tissue Resident Macrophage Differentiation During Nematode Infection of the Pleural Cavity. bioRxiv (2021). doi: 10.1101/2021.12.17.472661 PMC7616141 36948193

[38] Al-Qaoud KM Taubert A Zahner H Fleischer B Hoerauf A. Infection of BALB/c Mice With the Filarial Nematode Litomosoides Sigmodontis: Role of CD4+ T Cells in Controlling Larval Development. Infect Immun (1997) 65(6):2457–61. doi: 10.1128/iai.65.6.2457-2461.1997 PMC175343 9169791

[39] Ehrens A Lenz B Neumann A-L Giarrizzo S Reichwald JJ Frohberger SJ. Microfilariae Trigger Eosinophil Extracellular DNA Traps in a Dectin-1-Dependent Manner. Cell Rep (2021) 34(2):108621. doi: 10.1016/j.celrep.2020.108621 33440150

[40] Kumar V. Innate Lymphoid Cell and Adaptive Immune Cell Cross-Talk: A Talk Meant Not to Forget. J Leukoc Biol (2020) 108(1):397–417. doi: 10.1002/JLB.4MIR0420-500RRR 32557732

