# Supplementary material for: ILC2s Control Microfilaremia During Litomosoides sigmodontis Infection in Rag2-/- Mice
Source: Front Immunol. 2022 Jun 9;13:863663. doi: 10.3389/fimmu.2022.863663 (PMC9222899; doi:10.3389/fimmu.2022.863663)
Supplement: Supplementary file 1 [file DataSheet_1.pdf]

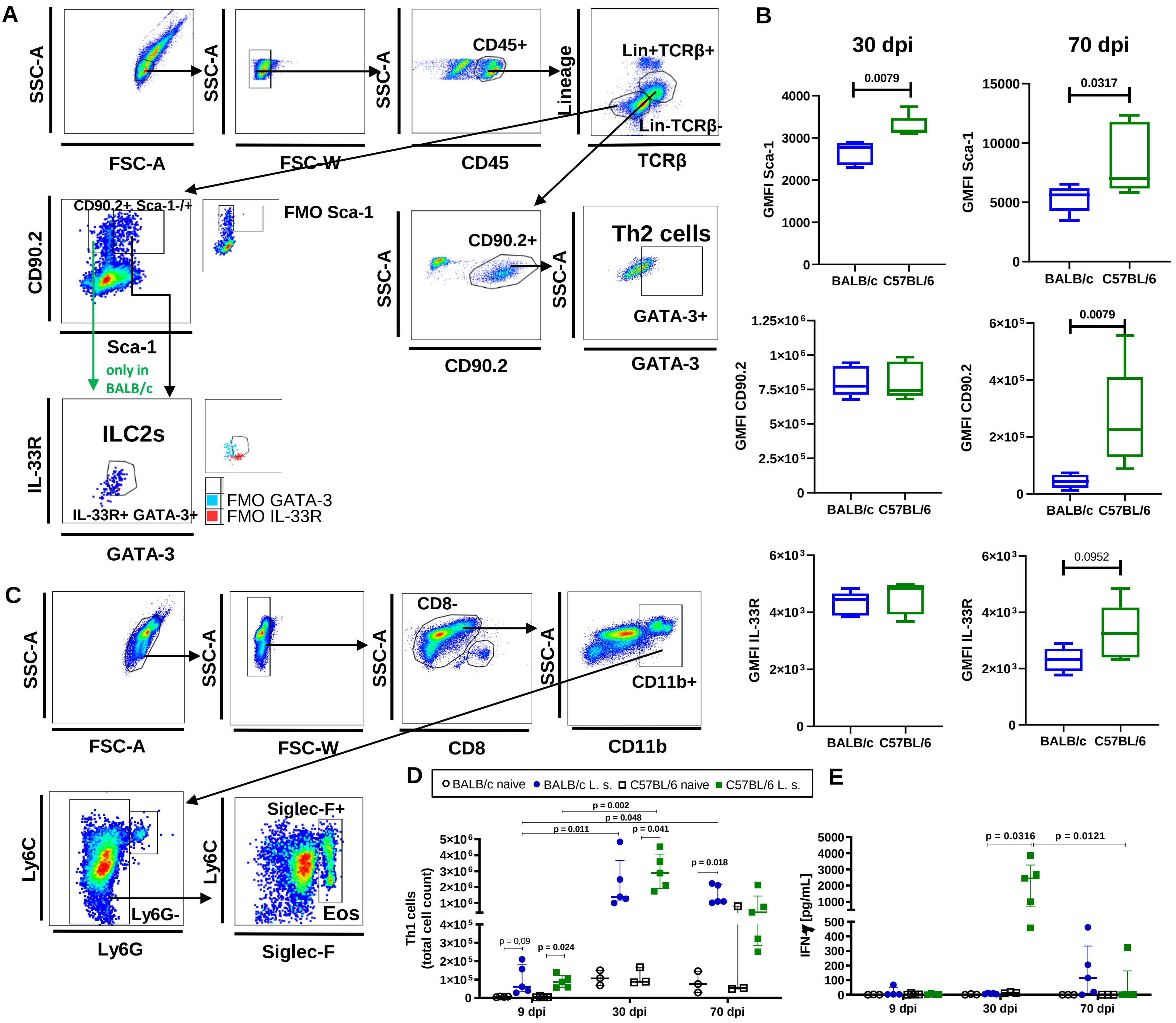

S1 Figure Gating strategies for ILC2s, Th2 cells and eosionphils as well as ILC2 phenotype. (A) Gating strategy to identify ILC2s and Th2 cells. ILC2s of BALB/c mice were identified as CD45^{+} Lin^{-} TCRβ^{-} CD90.2^{+} Sca-1$^{+/-}$ IL-33R^{+} GATA-3^{+}, ILC2s of C57BL/6 mice were identified as CD45^{+} Lin^{-} TCRβ^{-} CD90.2^{+} Sca-1^{+} IL-33R^{+} GATA-3^{+}. Th2 cells were identified as CD45^{+} Lin^{+} TCRb^{+} CD90.2^{+} GATA-3^{+} **(B)** Sca-1, CD90.2 and IL-33R expression (GMFI) of ILC2s 30 dpi (left panel) and 70 dpi (right panel). **(C)** Gating strategy to identify eosinophils. Eosinophils were identified as CD8^{-} CD11b^{+} Ly6C^{int-hi}, Ly6G^{-} Siglec-F^{+}. **(D)** Total cell count of Th1 cells (CD45^{+} Lin^{+} TCRb^{+} T-bet^{+}) in the pleural cavity. **(E)** IFN-γ levels in the pleural cavity lavage. n = 5, data are representative for 2 independent experiment per timepoint. Data shown as median with IQR; Kruskal-Wallis with Dunn's post-test.

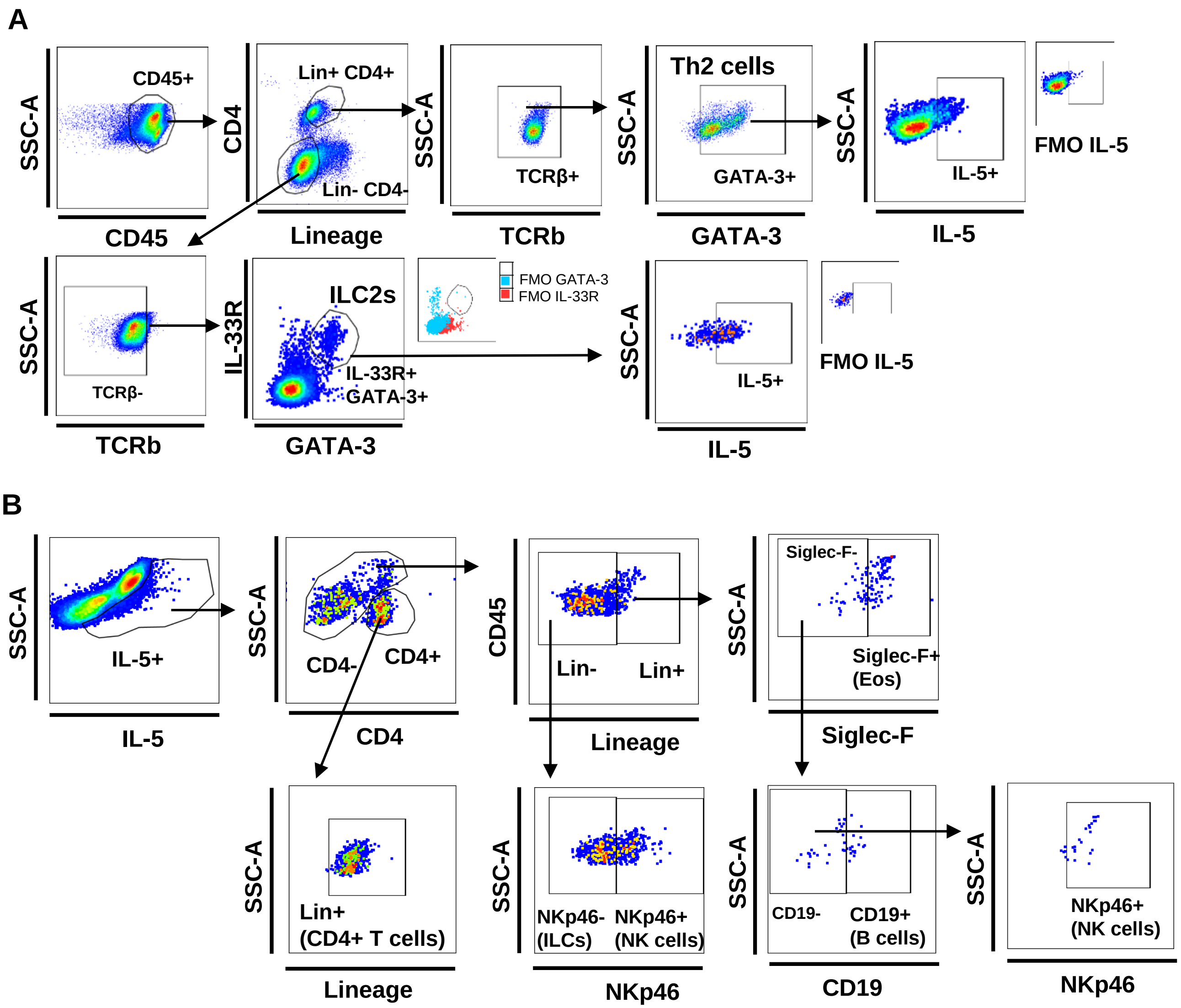

S2 Figure Gating strategies to identify IL-5+ cells. (A) Gating strategy to identify IL-5-expressing ILC2s and CD4^{+} T cells. IL-5-expressing ILC2s were identified as CD45^{+} Lin^{-} TCRβ^{-} CD4^{-} IL-33R^{+} GATA-3^{+} IL-5^{+} and IL-5-expressing Th2 cells were identified as CD45^{+} Lin^{+} CD4^{+} TCRβ^{+} GATA-3+ IL-5^{+}. **(B)** Gating strategy for analyzing the composition of IL-5+ cells in the pleural cavity. IL-5+ eosinophils were identified as IL-5+ CD4- Lin+ Siglec-F+. IL-5+ CD4+ T cells were identified as IL-5+ CD4+ Lin+. IL-5+ B cells were identified as IL-5+ CD4- Lin+ Siglec-F- CD19+. IL-5+ Lin+ NK cells were identified as IL-5+ CD4- Lin+ Siglec-F- CD19- NKp46+. IL-5+ Lin- NK cells were identified as IL-5+ CD4- Lin- NKp46+.

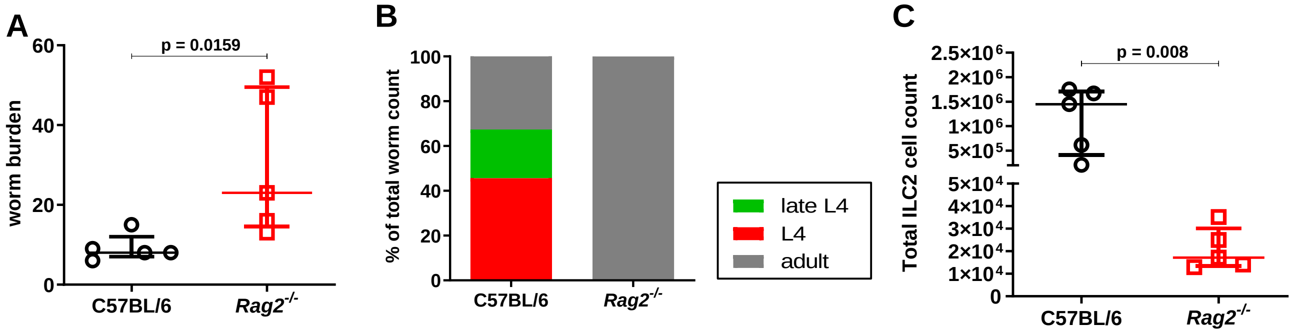

S3 Figure *Rag2*$^{-/-}$ show a significantly increased worm burden compared to C57BL/6 mice 30 dpi. (A) Worm burden 30 days post natural *L. sigmodontis* infection in semi-susceptible C57BL/6 mice and susceptible *Rag2*$^{-/-}$ mice. **(B)** Stages of worms 30 dpi. **(C)** Total cell count of ILC2s in the pleura 30 dpi. Bars represent the median with interquartile ranges. Data in A+C n = 5, data from 1 experiment. Data in B n = 42 worms for C57BL/6 mice, n = 146 worms for *Rag2*$^{-/-}$ mice, data from 1 experiment. Data were analysed with Mann-Whitney test, A p-value below 0.05 was considered to be significant.

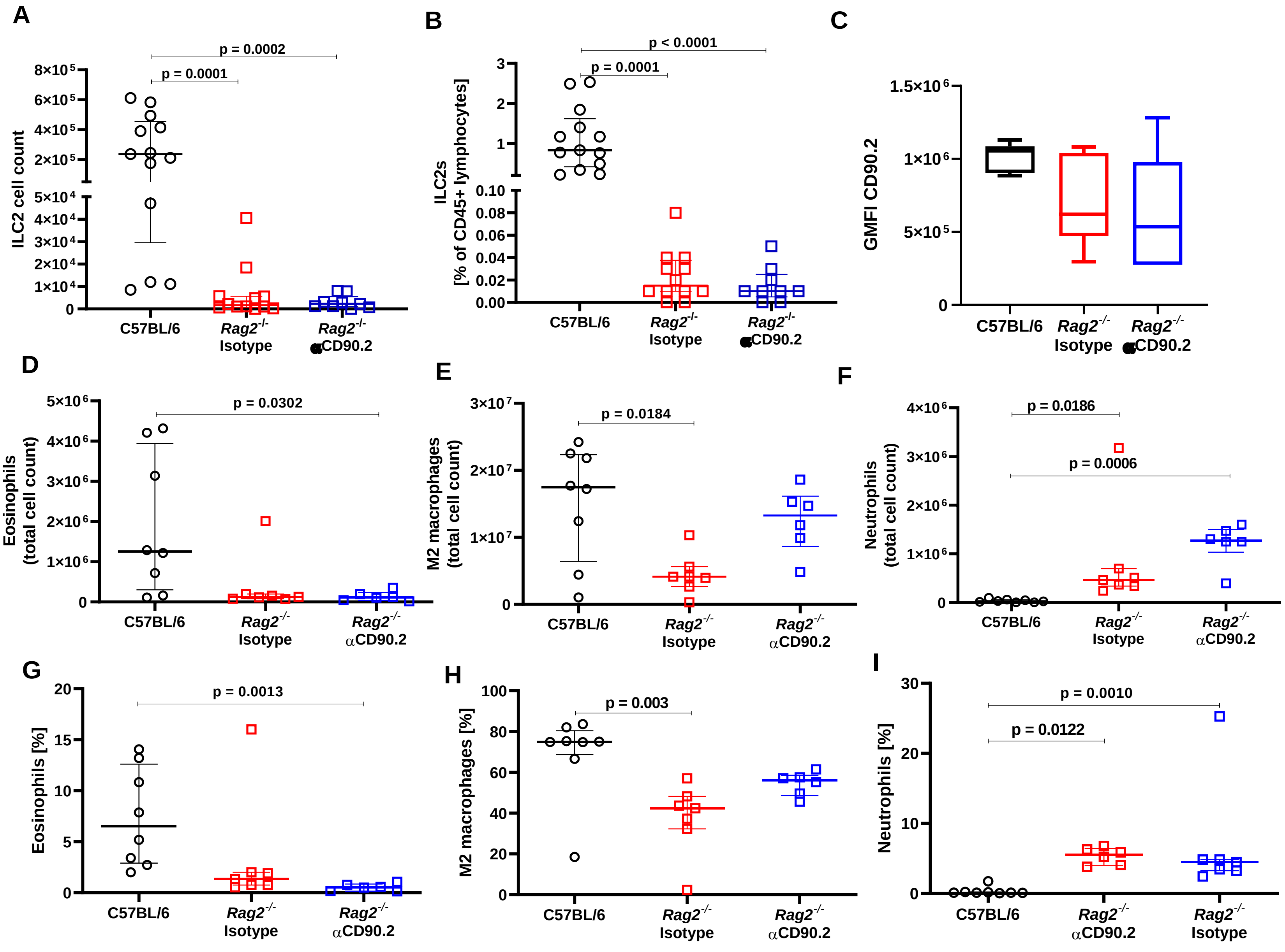

S4 Figure ILC2 depletion in *Rag2*$^{-/-}$ does not alter the myeloid cell composition in the pleural cavity upon *L. sigmodontis* infection. (A) ILC2 cell counts in the pleural cavity of naturally *L. sigmodontis*-infected C57BL/6, non-depleted and depleted *Rag2*$^{-/-}$ mice. **(B)** ILC2 proportion [% of CD45+ lymphocytes] in the pleural cavity. **(C)** CD90.2 expression (GMFI) of ILC2s in the pleural cavity. Total cell count of **(D)** eosinophils, **(E)** M2 macrophages, **(F)** neutrophils in the pleural cavity. Proportion [% of pleural cavity cells] of **(G)** eosinophils, **(H)** M2 macrophages and **(I)** neutrophils in the pleural cavity. Data shown as median with IQR. n=11-13, pooled data from 2 independent experiments. Kruskal-Wallis with Dunn's post-test.
